# SlypNet: Spikelet-based yield prediction of wheat using advanced plant phenotyping and computer vision techniques

**DOI:** 10.3389/fpls.2022.889853

**Published:** 2022-08-04

**Authors:** Arpan K. Maji, Sudeep Marwaha, Sudhir Kumar, Alka Arora, Viswanathan Chinnusamy, Shahnawazul Islam

**Affiliations:** ^1^Division of Computer Application, Indian Agricultural Statistics Research Institute, Indian Council of Agricultural Research, New Delhi, India; ^2^Division of Crop Physiology, Indian Agricultural Research Institute (ICAR), New Delhi, India

**Keywords:** deep learning, plant phenotyping, segmentation, spike detection, spikelet detection, yield prediction, SlypNet, Computer vision

## Abstract

The application of computer vision in agriculture has already contributed immensely to restructuring the existing field practices starting from the sowing to the harvesting. Among the different plant parts, the economic part, the yield, has the highest importance and becomes the ultimate goal for the farming community. It depends on many genetic and environmental factors, so this curiosity about knowing the yield brought several precise pre-harvest prediction methods using different ways. Out of those techniques, non-invasive yield prediction techniques using computer vision have been proved to be the most efficient and trusted platform. This study developed a novel methodology, called SlypNet, using advanced deep learning networks, i.e., Mask R-CNN and U-Net, which can extract various plant morphological features like spike and spikelet from the visual image of the wheat plant and provide a high-throughput yield estimate with great precision. Mask R-CNN outperformed previous networks in spike detection by its precise detection performance with a mean average precision (mAP) of 97.57%, a F1 score of 0.67, and an MCC of 0.91 by overcoming several natural field constraints like overlapping and background interference, variable resolution, and high bushiness of plants. The spikelet detection module’s accuracy and consistency were tested with about 99% validation accuracy of the model and the least error, i.e., a mean square error of 1.3 from a set of typical and complex views of wheat spikes. Spikelet yield cumulatively showed the probable production capability of each plant. Our method presents an integrated deep learning platform of spikelet-based yield prediction comprising spike and spikelet detection, leading to higher precision over the existing methods.

## Introduction

The agriculture sector has immensely contributed to the economy, from achieving food and energy security to accomplishing livelihood for a significant part of the population. Innovative computing technologies greatly help automation in the agriculture sector by reducing the burden of time-consuming, labor-intensive, and highly erroneous agricultural processes (AI for the farmer | The Indian Express). Digital and supercomputing systems, networking facilities, and efficient client interfaces have started to transform the whole agriculture sector.

Yield can be in different forms: grain, fiber, fodder, root, etc. It fulfills the purpose of consumption and the source of revenue for society. Innovative techniques are being significantly used in the farmers’ fields as well as in the breeding trials to increase the yield. Therefore, assessing the potential yield from the standing crop would be very helpful in improving cropping management (Greener et al., 2021), which generates the need for high-throughput plant phenotyping. Plant phenotyping is an emerging field in agricultural sciences that links plant genomics with plant physiology, ecology, and agronomy. Automated high-throughput plant phenotyping refers to the non-destructive sensing of plant images on a large scale and extracting many useful phenotyping features (Kumar et al., 2016). Plant’s anatomical and physiological traits, such as vigor, leaf area, biomass, and inflorescence structure, are the primary quantitative parameters throughout a crop’s life cycle. Nowadays, a deep and refined assessment of these traits has become a toolbox for plant breeders in choosing suitable genotypes for their specific field of research, e.g., abiotic stress tolerance, disease or pest resistance, and yield improvement (Hartmann et al., 2011; Ubbens and Stavness, 2017).

Several research works are available in the literature on non-invasive yield estimation using high-throughput plant phenotyping sensors. Some used auxiliary agrarian factors in assessing yield (Elavarasan et al., 2018), while others applied pioneer image processing (Qiongyan et al., 2017; Tan et al., 2020) and AI (artificial intelligence) technologies (Ullah et al., 2021) in laboratory or field conditions.

The quantification of the yield of experimental plants has always been the primary research area of crop scientists (Yoosefzadeh-Najafabadi et al., 2021). Various vegetation or growth traits have shown a significant correlation with the yield potentiality of plants (Ajlouni et al., 2020). They can be measured from different data sources like visual, hyperspectral data, thermal, and infrared. Besides the physiological parameters, the ecological factors, *viz.*, water availability, temperature, and other natural resources, also have the potential role in determining the crop yield (Elavarasan et al., 2018; van Klompenburg et al., 2020). Zhou and Soldat (2021) developed various models for these physiological and ecological factors and yields using many statistical and machine learning techniques. These factors analyze only the phenotypes, not the genotypic ability such as tolerance to stress, disease resistance, and dwarf nature. Consequently, plants with distinct canopy behavior can also have the same yield, leading to a potential bias in the yield estimates. Moreover, the yield estimation is also very complex and error-prone due to the dynamic nature and a large number of auxiliary factors.

In line with Qiongyan et al. (2014, 2017) and Narisetti et al. (2020), the other possible solution for yield estimation is the application of visual aids. Technology upgradation in the sensing process can cope with any field situation. The non-dependency on ecological factors and highly efficient image processing techniques always outperformed the existing models based on agrarian factors (Arya et al., 2022). Advanced neural network technologies like texture segmentation (patterns, photographs) in image partitioning (Qiongyan et al., 2014, 2017) and pixel segmentation on threshold values of plant objects (Tan et al., 2020) performed better and obtained a relatively good score in spike detection, but the color feature-based segmentation methods may generate false detection in some growth stages, e.g., between green spike and green leaves in the early reproductive stage of the plants.

The yield estimation can also be obtained through computer vision (Ullah et al., 2021). Evolution in computer vision provided us with various novel convolutional neural network (CNN) based deep learning architectures like single-shot detector (Zhao et al., 2021), Fast Region-based CNN (R-CNN), Faster R-CNN (Madec et al., 2019), You Only Look Once (YOLO) (Singh et al., 2021), U-Net (Misra et al., 2020), and Mask R-CNN (Johnson, 2018; He et al., 2020; Shu et al., 2020; Xu et al., 2020) and solved many visionary problems in agriculture like disease or pest detection and classification (Su et al., 2021), assessment of plant biomass (Misra et al., 2019), production forecasting (Elavarasan et al., 2018), remote sensing data analysis (Sagan et al., 2021), and crop yield estimation (van Klompenburg et al., 2020).

Yield means the measure of the revenue part of the crop plant. In the wheat crop, grain, the edible part, is present within the spikelet of the ear or spike of the plant (inflorescence). Wheat spike has distinguishable features and zigzag orientation of spikelets (Ajlouni et al., 2020). Existing studies on the prediction of plant yield using object detection techniques are illustrated mainly in two ways. First, only the spike detection was used to predict the yield, and second detecting and counting spikelets from the whole plant image was used to predict the yield. Spike detection by existing object detection models provides different accuracy with certain constraints. R-CNN models of Hasan et al. (2018) achieved an average detection accuracy ranging from 88 to 94% despite the challenging field imaging conditions, e.g., variable illumination conditions, high spike occlusion, and complex background. Misra et al. (2020, 2021) developed an advanced hour-glass fitted U-Net model, SpikeSegNet, for spike segmentation with a high precision (accuracy of about 99%) but faced problems like overlapping spikes and required additional counting algorithm. SSR-Net (Wang et al., 2021), a multistage convolutional neural network, counted wheat ears with 98% accuracy, and Zhao et al. (2021) used unmanned aerial vehicle (UAV) images to detect wheat spikes using an improved yolov5 model (about 94% accuracy). Other improved object detection algorithms proposed in the literature for the agricultural crop are TasselNetv2 (Xiong et al., 2019), a context-augmented CNN-based local regression network for in-field counting of maize tassels (91% accuracy) and fruit detection in strawberry by Yu et al. (2019) using Mask R-CNN (average precision of about 96%). These reviews of the literature on spike detection models state that existing models resulted in good accuracy but faced typical constraints like overlapping spikes, low and variable resolution, background, leaf interference, and variable illumination that motivated to redesign the spike detection model with high precision and more adaptability to the constraints.

Although, in the wheat crop, the spike number serves as the basis of yield prediction (Tan et al., 2020), all spikes cannot show identical features (spikelet number varies from plant to plant). As reported by Alkhudaydi and Zhou (2019), a robust yield estimation always depends on the spikelet, which is a more exact feature than a spike. SpikeletFCN of Alkhudaydi and Zhou (2019), a VGG16-based FCN, made spikelet counting with high-resolution images and reduced the error to 89%. This direct approach improved the spikelet counting accuracy. However, it failed in low-resolution images, undercounting spikelets when all spikelets were not visible in dense vegetation, and overcounting spikelets in diseased plants with identical (with spikelet) spots.

Besides the existing methodologies like spike-based yield prediction and direct spikelet counting approaches, a novel two-staged methodology for yield prediction is proposed. In this methodology, a spike detection followed by a spikelet detection technique is developed with the spikelet density (average spikelet number in spikes) as the main quantitative parameter for the yield prediction.

## Materials and methodology

**Figure 1:**
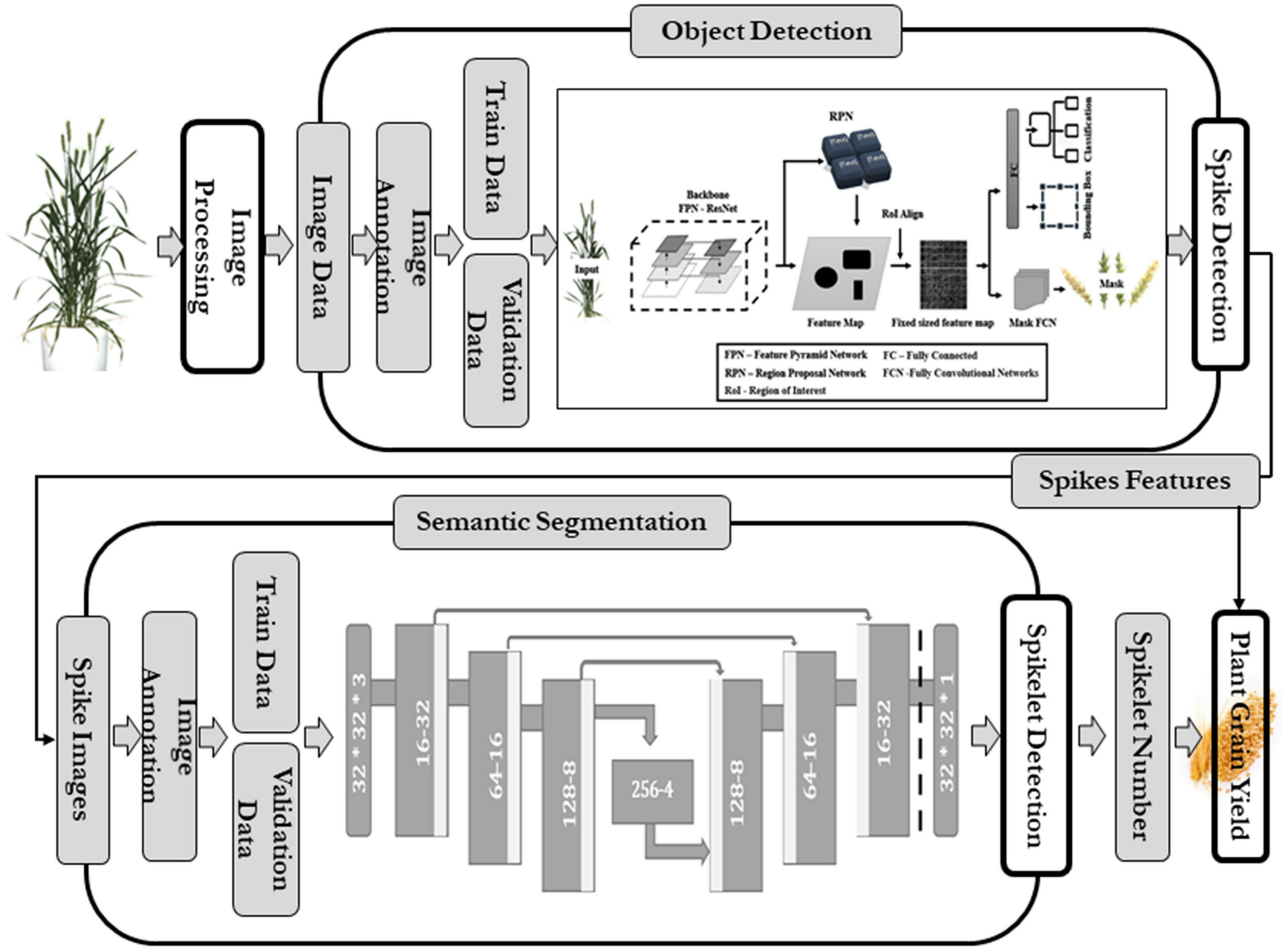
Flowchart of current methodology.

In SlypNet, a new object detection network is presented that can detect spikes from the images of wheat plants with great precision by overcoming the constraints like overlapping spikes, low and variable resolution, background and leaf interference, and variable illumination. Following that, another deep learning network performs the segmentation of the spikelets from each spike, and later, an explicit method predicts the spikelet-based yield using the extracted plant features (Figure 1).

### Growing of plants and image acquisition

**Figure 2:**
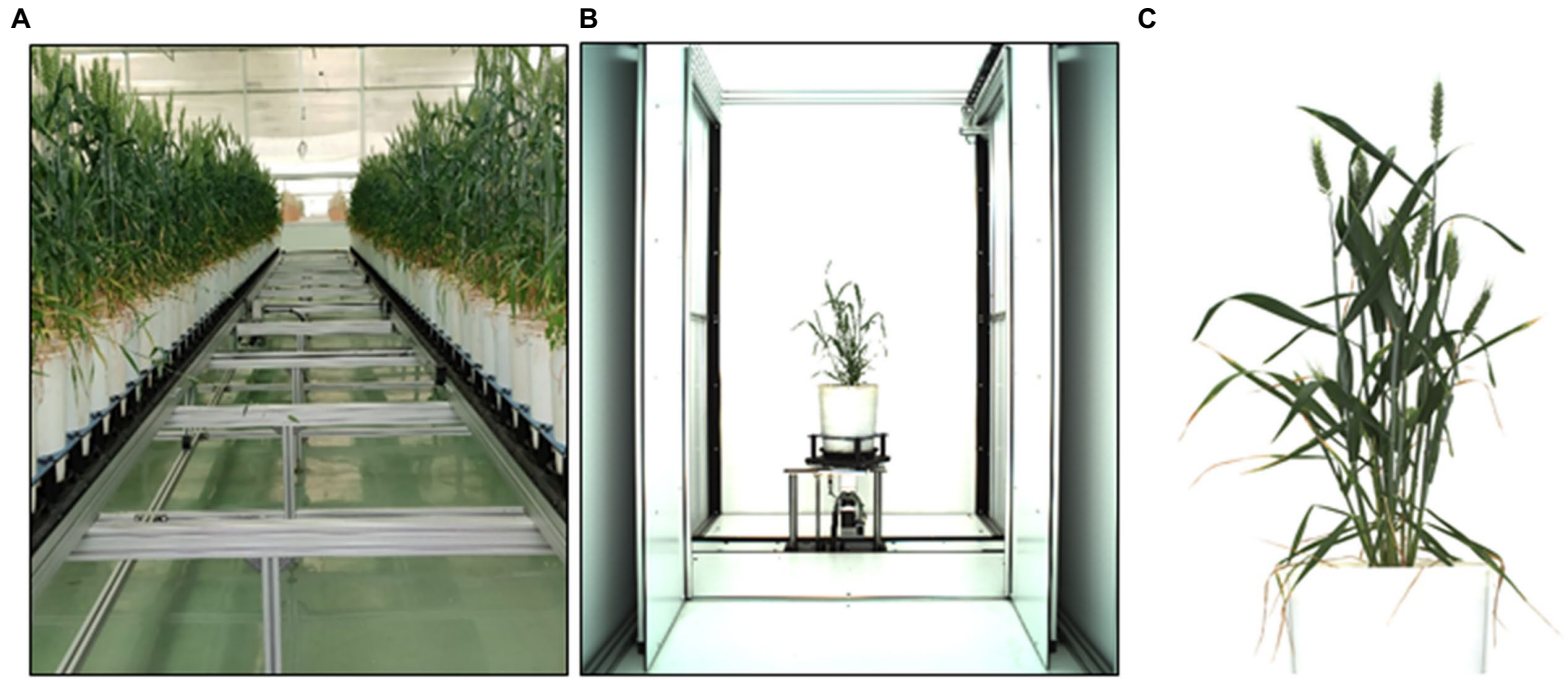
**(A)** Wheat plants grown under greenhouse, **(B)** the imaging chamber, and **(C)** cropped plant image.

A wheat experiment was conducted during 2017–2018 in the climate-controlled greenhouse of Nanaji Deshmukh Plant Phenomics center located in the north-western part of New Delhi, India (mean sea level—228.61 meters) (Figure 2A). Pots with the dimension of 0.19 m diameter, 0.4 m height, and 15-liter volume filled with 12.5 Kg uniformly mixed soil per pot were used for sowing the experimental material (Seed). Under natural light illuminated conditions, the experiment was conducted with a controlled sinusoidal temperature of 24°C (day time) and 16°C (night time) inside the greenhouse. Relative humidity (RH) between 50 and 60% was maintained using an additive humidifier whenever required. The sowing was carried out during the last fortnight of 26 November 2018 and awaited harvesting until the plant attained physiological maturity during the second fortnight of 10 April 2019. Spikes started emerging in tillers from the mid of February, and fully grown spikes and spikelets started being observed from the end of February. For acquiring the image dataset, a set of 1200 wheat plants was subjected to a sensor (300 plants per day, so that one set took four consecutive days) under greenhouse conditions. In this study, imaging was done three times (3600 images), (first on 1, 2, 3, and 4 of March and another two on 9, 10, 11, and 12 of March and 26, 27, 28, and 29 of March) and obtained a mixed dataset containing images of all types of the plant starting from young green plants to yellow matured plants.

A uniform white background was maintained to increase separation accuracy between background and plant parts. The visual imaging required a highly efficient camera (Prosilica GT 6600), a spectral band of visual range (400–700 nm), and a sensor (6576 × 4384 pixels) on semiconductor KAI-29050 (AVT Prosilica GT 6600—Vital Vision Technology Pte Ltd). The peak reflectance was around 550 nm, and the low absorbance of the pigments in this wavelength region made plants look green and enabled us to detect plant tissue in RGB images. Three different side view images (angles: 0°, 120°, and 240°) of the plants were recorded using the automated turning and lifting unit present inside the imaging unit. The side views were considered (Figure 2B) as it was hypothesized that the image from one direction could not cover all the spikes of a plant; besides, it helped increase the data points corresponding to one plant. Analyzing visible light images delivered information about dimensions, morphological and geometric properties, and color distributions (Das et al., 2017).

### Spike detection algorithm

The spike detection module extracted spikes from plant images using an object detection algorithm. This study evaluated existing methods using computer vision, and improvement cues were taken. Advanced R-CNN models (Hasan et al., 2018; Madec et al., 2019) could provide good precision in spike detection using bounding boxes, while U-Net models create pixel-to-pixel translation and detect object pixels with higher accuracy (Misra et al., 2020). Both faced certain constraints (overlapping problems led to undercounting of spikes, the additional need of counting algorithm in U-Net, R-CNN gave only bounding boxes, not exact object pixels) in this spike detection. Therefore, the latest object detection network of R-CNN, Mask R-CNN, was used, and the image dataset was passed for training and to develop a data-specific Mask R-CNN model of spike detection. The details of this module are given in Figure 1.

#### General network representation of Mask R-CNN

**Figure 3:**
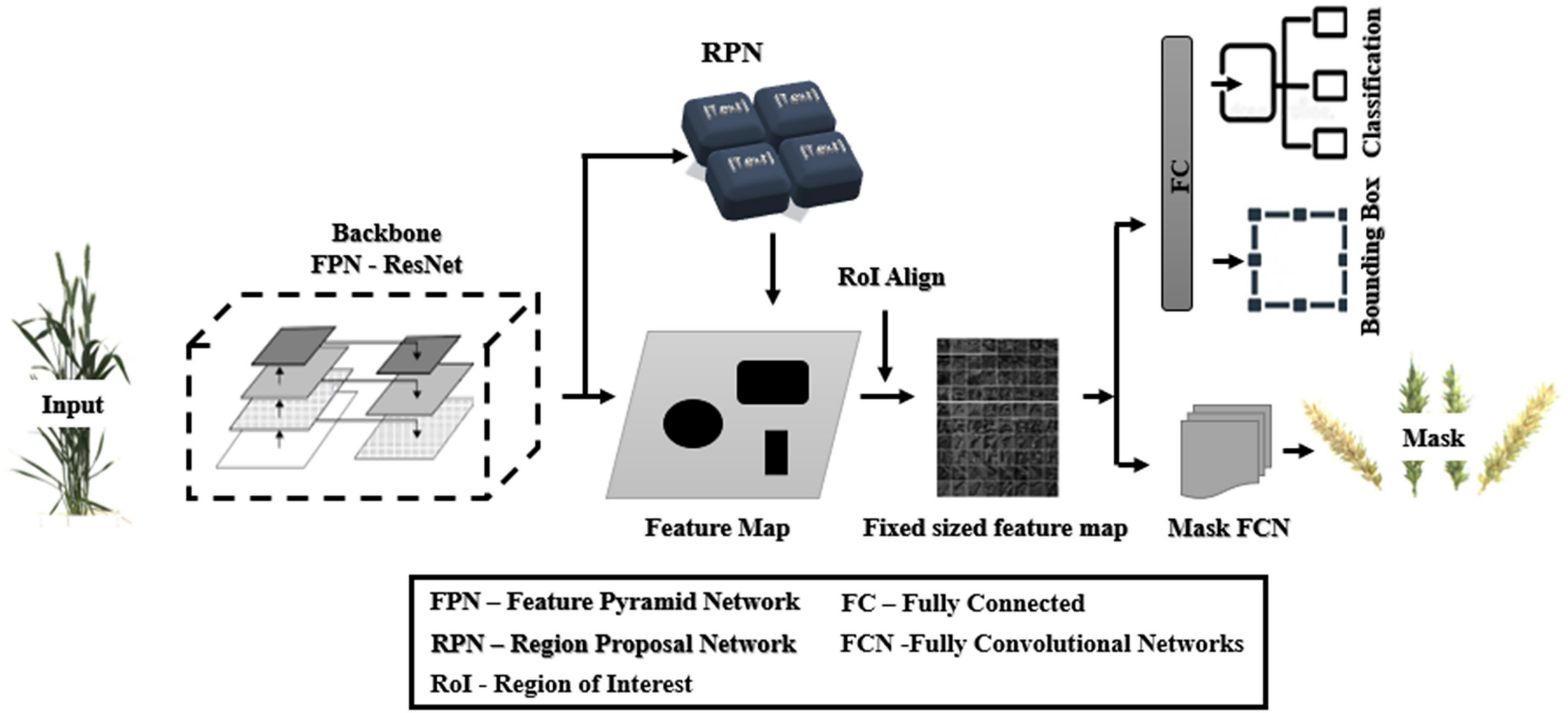
General architecture of Mask RCNN (redrawn from He et al., 2020).

Faster R-CNN was extended into Mask R-CNN with the addition of a branch to predict a high-quality segmentation mask for objects in parallel with the existing branch for bounding box recognition and classification (Figure 3; He et al., 2020). It is the latest state-of-the-art algorithm for object detection comprising the localization, classification, and instance segmentation of objects in the images and has been used in several studies, *viz.*, automatic segmentation of microscopy images of cell nuclei (Johnson, 2018), cattle counting (Xu et al., 2020), multiorgan segmentation (Shu et al., 2020), service robot’s target capture task (Shi et al., 2019), and for the detection of *Fusarium* Head Blight in agriculture (Su et al., 2021). It has two units (Fan et al., 2020): the first unit generates proposals for the regions with or without objects from the input image and, in contrast, the second one predicts the object’s class, refines the bounding box, and generates a pixel-level mask based on the first stage proposals. Both units are connected with the basic backbone structure of the Feature Pyramid Network (FPN).

#### Training dataset

While capturing the plant images, some parts of the imaging chamber also came up in the picture. So, each image in the acquired dataset was cleaned using an optimistic image cropping, and also, a particular dimension of 3072 × 2048 was maintained (Figure 2C). The shape defined here was a multiple of 2^6^ required for a convolutional network (He et al., 2020). The spike annotation was done with one class as “spike” on an online interface (Supplementary Figure S1) (VGG Image Annotator, November 15, 2021) and saved as a JSON file for training and validation datasets (Table 1) (train test split with test size 0.2).

**Table 1:** Basic training configuration of mask R-CNN.

SL No.	Arguments	Values
1	Image_Min_Dim	800
2	Image_Max_Dim	3100
3	Learning_Rate	0.001
4	Learning_Momentum	0.9
5	Weight_Decay	0.0001
6	Steps_Per_Epoch	100
7	Detection_Min_Confidence	0.9
8	Rpn_Anchor_Scales	[32, 64, 128, 256, 512]
9	Images_Per_Gpu	1
10	Num_Classes	1+1 (“Spike,” “Background”)
11	Training and Validation Data	2000 and 500 (train-test-split with test size 0.2)
12	Testing Data	Varies
13	Epochs	100

#### Updated skeleton and parameters of the network

The training parameters of the network were set according to the study requirement (Table 1). Besides these basic parameters, the network underwent several structural changes to improve the precision of the images with underlying constraints (overlapping spikes, low and variable resolution, background and leaf interference, variable illumination). Every training was performed in two successive phases for better model performance. The following modifications were adopted in the spike detection model—(a) backbone “ResNet” network was configured as “ResNet50” or “ResNet101” with 5, 22 layers of residual blocks, respectively (He et al., 2020), (b) network’s backbone built up with two types of convolutions, i.e., normal or separable convolution (Chollet, 2017), and (c) input data were taken as RGB or HLS (using the OpenCV-python library). While training, the dataset was augmented using certain augmentation techniques (python imgaug library), *viz.*, horizontal flip, vertical flip, rotation (angle 30^0^), scaling (80 to 120% size of the images), add (add a value to all pixels in range, [−40, 40]), multiply (multiply all pixels with a random value in a specific range, [0.8, 1.5]), and Gaussian blur (blur images using Gaussian kernels of size 5).

#### Evaluation of spike detection network

Mask R-CNN as the extended form of Faster R-CNN performs object detection comprising the classification of the objects and the creation of bounding boxes. In addition, the extra mask unit makes segmentation of the foreground from the background of objects. In this study, the network would detect only one type or class of objects, “Spike,” so all the objects detected in one image would spike only. As the network did one classed detection, the evaluation of the classification performance of the network was avoided. The primary evaluation was done for the performance of localization, i.e., bounding box and mask. The proposed architectures of Mask R-CNN were evaluated with various quantitative metrics, i.e., loss functions obtained at training, mostly used object detection metrics, i.e., using spatial overlaps (precision, recall, mean average precision, F1 score, and Matthews correlation coefficient (MCC)) for binary segmentation of spikes. Except for loss, a higher value of all metrics shows better network performance than the others.

The validation loss functions were good evaluation metrics for the bounding box and the mask unit in Mask R-CNN (He et al., 2020). Those were Smooth L1 loss for the bounding box and binary cross-entropy or log loss for the mask. Smooth_L1_loss (Equation 1) is less sensitive to outliers than mean square error (mSE) and, in some cases, prevents exploding gradients (Girshick, 2015).


li=0.5∗di2β  if di<βdi−0.5∗βotherwise

βspecifies the threshold,non−negative,the default value is1.0

(1) $$\mathrm{smooth}l1\;\left(x,y\right)=L={\left\{l1,\ldots ,lN\right\}}^{T}$$

Tdenotes reduction,that is “none”, “mean”and “sum”.

Binary cross-entropy or log loss (Equation 2) compares each predicted probability to the actual class output, which can be either 0 or 1. Since the log of values between 0.0 and 1.0 is negative, it takes the negative log to obtain a positive value for the loss. When the predicted probability of the true class gets closer to zero, the loss increases exponentially (Misra et al., 2020; Supplementary Figure S2).


(2) $$\begin{array}{l} \mathrm{bin}\_\mathrm{cross}\_\mathrm{entropy}=\frac{1}{n}\overset{n}{\underset{i=1}{\sum }}y_{i}*\log x_{i}\\ \;\;\;+\left(1-y_{i}\;\right)*\log\;\left(1-x_{i}\right) \end{array}$$

$$\begin{array}{l} \mathrm{where},\;x,\;y,d,\mathrm{and}\;n\;\mathrm{is model output},\;\mathrm{target output},\\ \mathrm{difference and number of scalar values in output},\;\\ \mathrm{respectively}. \end{array}$$

After loss function, networks were further evaluated by the most popular evaluation metric in the case of object detections, the average precision (AP). As the detection also performs the classification of detected objects, other metrics like F1 score and MCC have also proved to be efficient in choosing the best classifier (Chicco and Jurman, 2020). This study primarily focused on the precision of localization of objects and binary segmentation of the object with only one class. The bounding box drew the region of interest with four coordinates, and the masking unit gave the binary mask of objects. The predicted mask of objects provides two values of pixels, i.e., the value 1 if it belongs to the object (foreground), and 0 depicts the background pixel of objects. Instead of classification, F1 and MCC measured the localization accuracy of different models. All these metrics used the spatial overlaps, IoU (also known as Jaccard) (Liu and Li, 2018), that finds the difference between ground truth and predicted coordinates of object mask within each bounding box. It computed IoU threshold value as 0.5, true detection (IoU ≥ 0.5), true positive (TP), wrong detection (IoU < 0.5), false positive (FP), fails to detect the object of class, false negative (FN) and true negative (TN) not detected non-object pixels. Precision, Recalls of each object’s values in each image were calculated (Equation 3).


$$\begin{array}{l} \mathrm{overlaps or Intersection over Union}\left(\mathrm{IoU}\right)\\ =\frac{pred_{\mathrm{mask}}\cap gt_{\mathrm{mask}}}{pred_{\mathrm{mask}}\cup gt_{\mathrm{mask}}} \end{array}$$

(3) $$\mathrm{precision}=\frac{\mathrm{TP}}{\mathrm{TP}+\mathrm{FP}},\mathrm{recall}=\frac{\mathrm{TP}}{\mathrm{TP}+\mathrm{FN}}$$

Precision is the ability of a model to detect relevant objects only, and recall measures the ability of the model to predict all ground truths correctly. Mask R-CNN calculates interpolated precision as average precision measured at 11 equally spaced recall levels of 0.0, 0.1, 0.2, … 0.9, and 1.0 from a monotonically decreasing PR graph (sorted recall) (Everingham et al., 2009). If the graph does not show this property, the graph needs to set a maximum precision for a recall value, i.e., the maximum to the right of that recall level, i.e., the maximum of all future points. Average precision (AP) was calculated as the arithmetic mean of the interpolated precision at each recall level (He et al., 2020) (Equation 4). A mean of APs (mAP) was computed for each image from the test dataset.


(4) $$\mathrm{AP}=\frac{1}{11}\;\underset{\mathrm{recall}}{\sum }{\mathrm{precision}}_{\mathrm{interpreted}}\left(\mathrm{recall}\right)$$

Besides mAP, another evaluation metric, the F1 score, could be obtained as a harmonic mean of derived mean precision and mean recall in each test image (Equation 5).


$$\mathrm{mPrecision}=\frac{1}{n}\overset{\mathrm{spikes}\;}{\underset{i}{\sum }}{\mathrm{precision}}_{i},\;\;\mathrm{mRecall}=\frac{1}{n}\overset{\mathrm{spikes}\;}{\underset{i}{\sum }}{\mathrm{recall}}_{i}$$

(5) $$F_{1}=\frac{2}{{\mathrm{mPrecision}}^{-1}+{\mathrm{mRecall}}^{-1}}$$

MCC is a more reliable statistical rate that only produces a high score if the prediction obtained good results in all our confusion matrix categories (true positives, false negatives, true negatives, and false positives) (Abhishek and Hamarneh, 2021).


(6) $$\begin{array}{l} {\mathrm{MCC}}_{\mathrm{mask}}\\ =\frac{\left(\mathrm{TP}*\mathrm{TN}-\mathrm{FP}*\mathrm{FN}\right)}{\sqrt{\left(\left(\mathrm{TP}+\mathrm{FP}\right)*\left(\mathrm{TP}+\mathrm{FN}\right)*\left(\mathrm{TN}+\mathrm{FP}\right)*\left(\mathrm{TN}+\mathrm{FN}\right)\right)}} \end{array}$$

Unlike mentioned metrics, MCC required TN also. It is mainly used for choosing the best classifier for object detection problems. However, in this segmentation study, the MCC was derived over binary masks of the objects (Equation 6). TN was taken as the whole image minus the union region of the predicted and ground truth mask (Zhang et al., 2016; Liu and Li, 2018; Supplementary Figure S3).

#### Output of spike detection network

The spike detection network resulted in segmented masks and bounding boxes of the spikes. The bounding box cropped the plant image, and the mask made a true image of the spike. Each spike image was used in the following deep learning network, i.e., spikelet detection, while the number of spikes (the count of bounding boxes) and the spike pixel area (the number of pixels within the spike) were passed for the yield prediction.

### Spikelet detection algorithm

Spikelet is the key component of the wheat plant for yield estimation. Therefore, it is a prerequisite to obtaining the total number of spikelets (present in a zigzag pattern) in each spike. Mostly semantic segmentation (U-Nets and FCNs) is used for this type of object detection (Ozturk et al., 2020), and at the same time, in the case of using small dimensional input data, U-Net performed better than FCN. Therefore, a set of architectures of U-Net models were used to detect the spikelet in the spikes.

#### General network representation of U-nets

**Figure 4:**
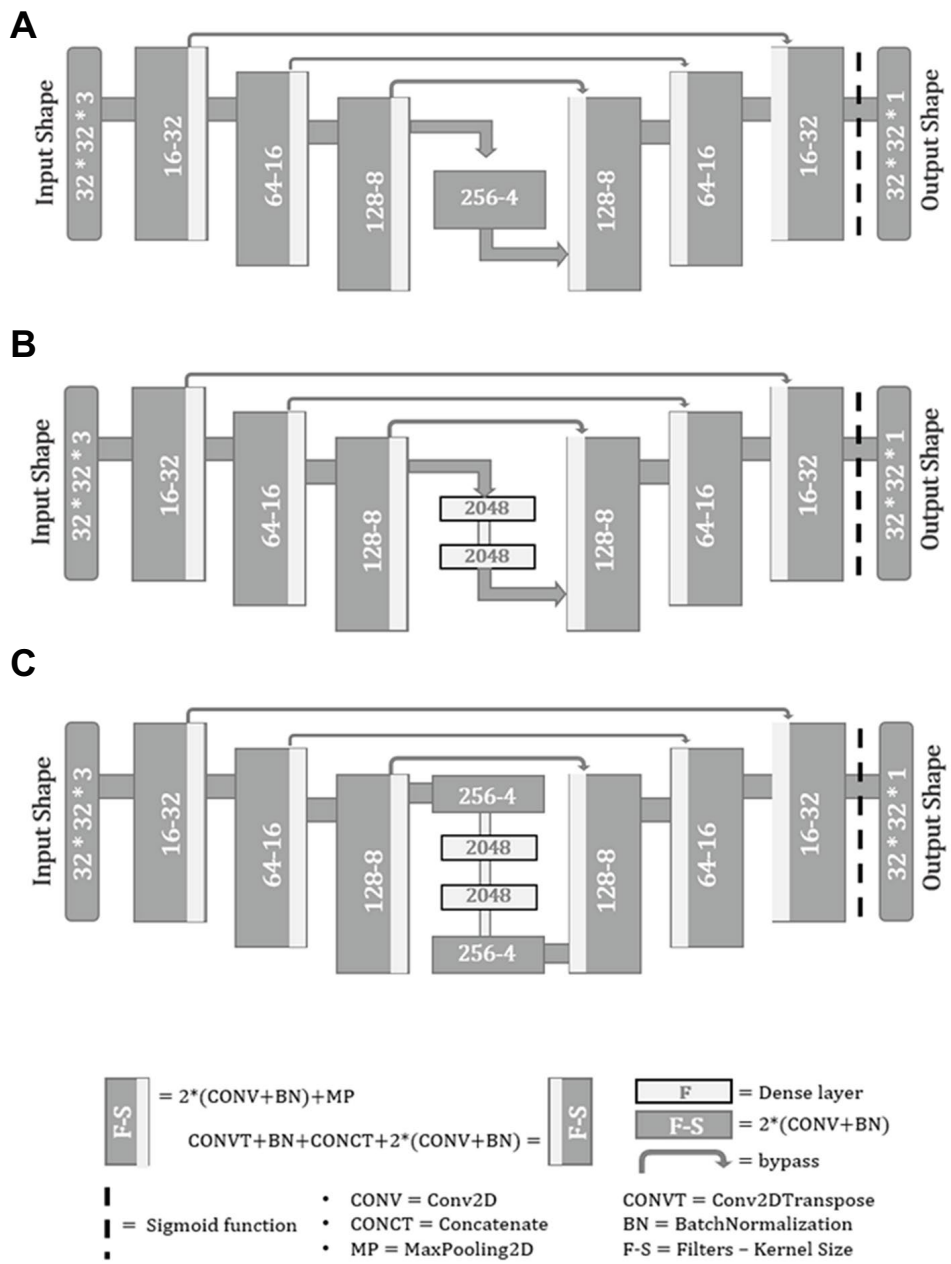
Spikelet detection networks.

Misra et al. (2020) presented highly efficient architectures for segmentation with advanced bottleneck network in U-Net models. Therefore, in this study, following U-Nets architecture was chosen for spikelet detection—(a) U-Net + 2 * Conv2D, (b) U-Net + 2 * Dense, (c) U-Net + 2 * Conv2D + 2 * Dense (Figure 4).

#### Training dataset

However, the 2D spike images had different orientations and distinct views of the spikelet, constraining the spikelet detection (Supplementary Figure S4) so that spike images with detectable spikelets should be subjected to model training as all spike images could not provide good segmentation. The spike detection module ran over a sample dataset of plant images provided the spike, out of which 250 images were passed as the training dataset of spikelet detection models (240 spike images (Supplementary Figure S5a) for training and 10 images for testing). As the ground truth, spikes were annotated to binary images based on specific morphological parts of spikelets using image processing algorithms (Supplementary Figure S5b). Both original and annotated images of spike were fragmented into overlapping patches (12015 patches) of size 32 × 32, and models were trained using the random train/test split method (test size = 0.2) (Misra et al., 2020).

#### Evaluation of spikelet detection module

U-Nets perform pixel-wise binary segmentation of objects. The log loss (Equation 2) is the most popular loss function in binary segmentation. The validation accuracy obtained from each training model helped evaluate the proposed networks well (Misra et al., 2020).

Besides that, further evaluation of the performance of the trained models took the output images of the spikes and measured additional three metrics, i.e., the number of over counting (OC) (detected spikelet number greater than the ground truth) and the number of undercounting (UC) (detected number less than the ground truth) and mean square error (mSE) (Equation 7) between the predicted number of spikelets and the ground truth number of spikelets from the test dataset of spikes.


(7) $$mSE=\frac{1}{n}\;\underset{n}{\sum }{\left(y_{i}-x_{i}\right)}^{2}$$

Where $y_{i}$ is the model output, $x_{i}$ is the target output, and n is the number of spikes.

The last three metrics mostly depict the adaptability of the models in the case of various constraint images of spikes and the ability to detect spikelets from them precisely.

#### Outputs of the spikelet detection network

Each patch of the spike went through binary segmentation, located the spikelets, and presented as binary dots. All these patches were used to recreate the full image of the spike (binary image). Using computer graphics, an optimized spikelet counting algorithm was designed to get an accurate number of spikelets from the binary images of spikes. The number of spikelets calculated from each plant spike was utilized in yield prediction.

### Spikelet yield prediction

In the final module, the spikelet-based yield prediction took all the quantified features obtained from spike detection and spikelet detection and derived the spikelet yield. As all the spike images do not show detectable representation of spikelets, the cumulative value of the detected spikelets might score false yield of a plant so that a better parameter, spikelet density that significantly relates to the genetic potentiality of spikelet production in each spike of a plant can be useful for the yield prediction. The computation of this density requires spikelet number and the vigor of spikes. Vigor can be represented in two ways, using the length of the spike and the pixel area of the spike. Later, one gives a better measure of spike vigor as the length led to complex and erroneous computation in the case of non-erected spikes.

The number of spikes, pixel area, and the number of spikelets were passed to the proposed equation of yield prediction (Equation 8) and obtained spikelet-based yield (SY) of the plant.


(8) SY=maxnum_spikeletipixel_areai ∗maxpixel_areai∗num_spike

## Results

SlypNet provided the path of yield prediction from plant images using deep learning models and image processing tools. Applied models were evaluated on certain test datasets with suitable metrics.

### Evaluation of spike detection networks

**Figure 5:**
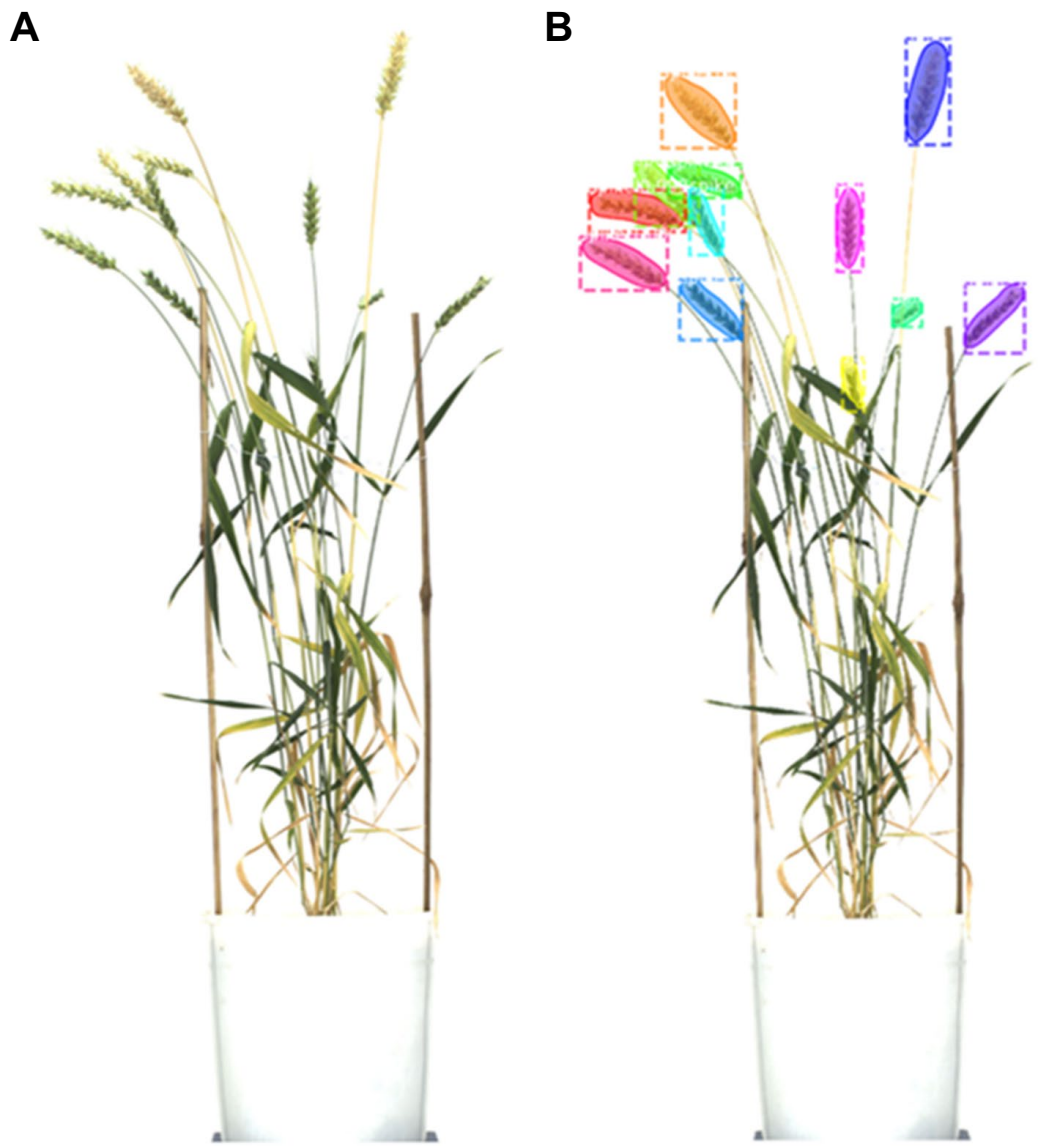
Result of the spike detection, **(A)** plant image, and **(B)** the detected spikes.

Performances of different architectures of Mask R-CNN in spike detection (Figure 5) were evaluated based on the following types of metrics.

#### Augmentation

**Figure 6:**
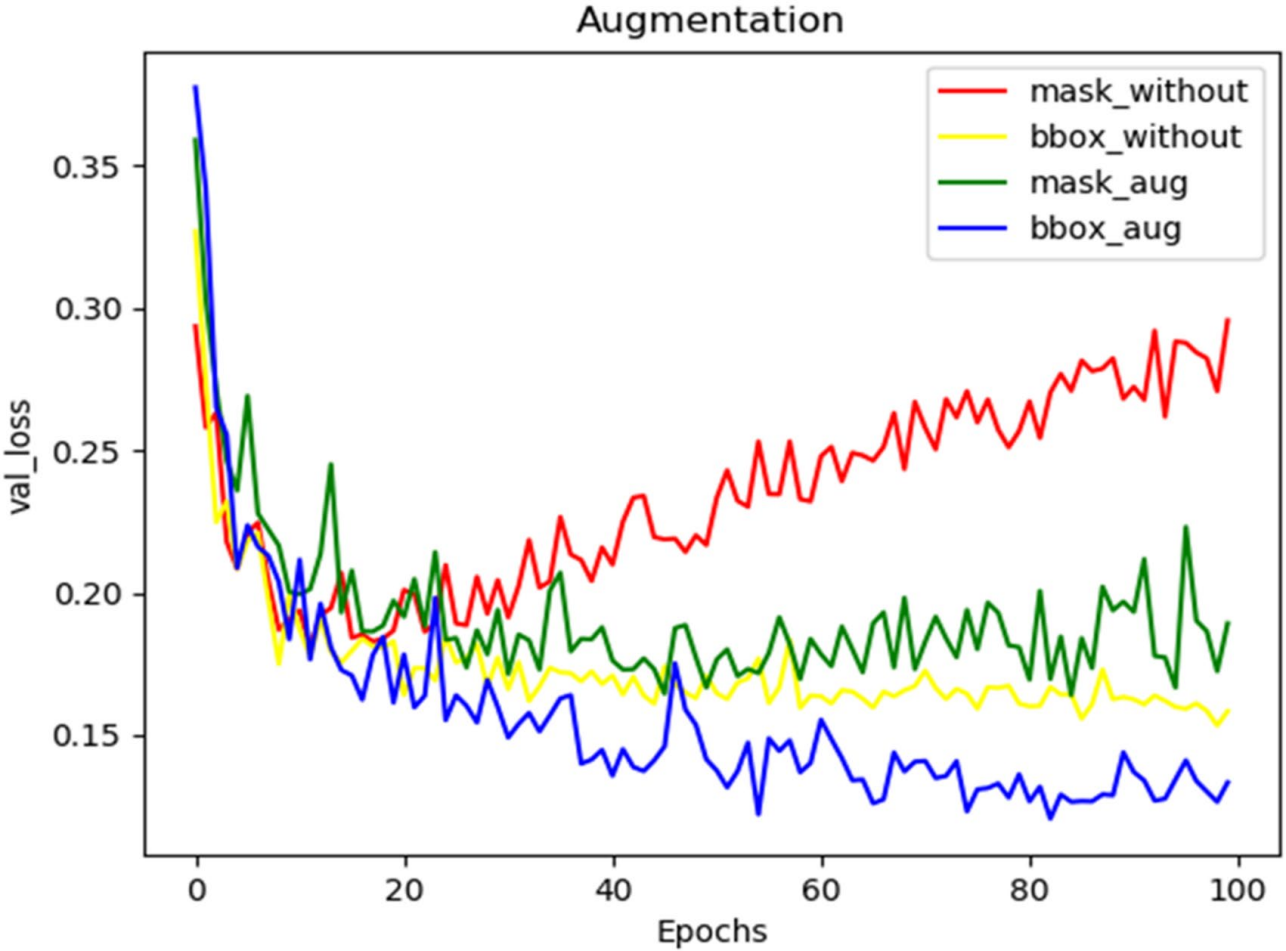
The validation loss curves of mask and bounding boxes from training the network without augmentation and with augmentation.

The effect of augmentation while training the spike detection model was depicted using the curves of validation losses (bounding box and mask) (Figure 6). Augmentation in training added noises to data and increased the size of the input dataset three times. Thus, overfitting in training models got reduced, and the model performance was enhanced.

#### Backbone network

For Mask R-CNN models, trained with two types of backbone networks (a) ResNet50, (b) ResNet101, and (c) ResNet50 + Separable Convolution (SepConv), the precision metrics were drawn using a test dataset of 24 images (mixture of plant images of different types, such as bushy, sparse, and with leaf interference).

**Figure 7:**
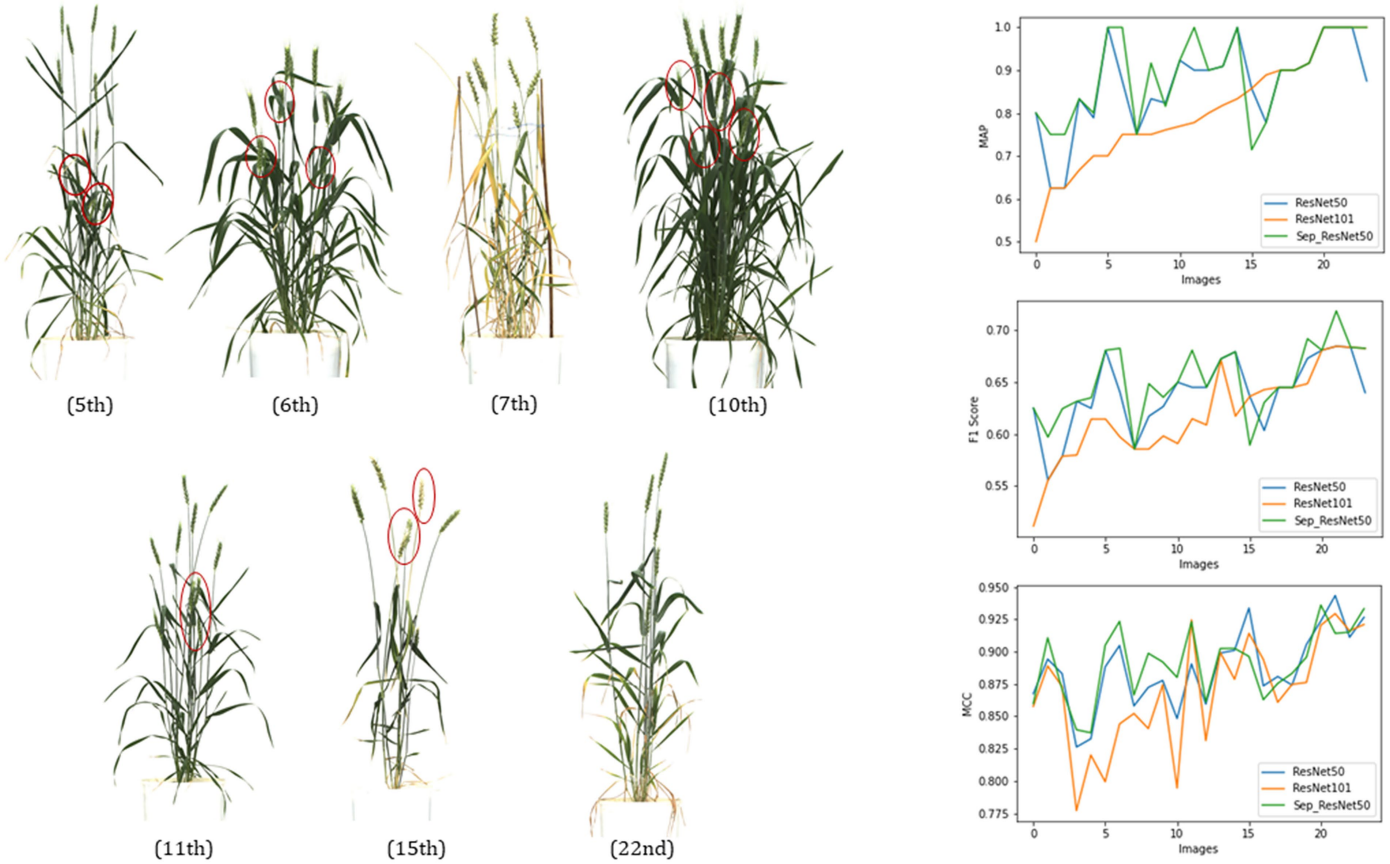
Evaluation of three networks, ResNet50, ResNet101, and ResNet50 + SepConv, of Mask RCNN for spike detection with metrics, mAP, Fl score, and MCC.

The metrics, mAP, F1, and MCC in the result (Figure 7; Table 2) showed that this single class object detection model of Mask R-CNN obtained good precision in light networks where the dense network lacked some precision in most of the images for a certain fixed size of training dataset and fixed number of iterations.

**Table 2:** Evaluation metrics of different trained models [ResNet50, ResNet101, and ResNet50 + Separable Convolution (SepConv)].

Network configuration	Test plants								
	*a*	*b*	*c*	*d*	*e*	*f*	*g*	Mean	Inference time (s)
	Average precision (AP) of plants							mAP	
ResNet50	1.0	0.875	0.75	0.923	0.899	0.857	1.0	0.8672	~1.92
ResNet101	0.699	0.75	0.75	0.769	0.777	0.857	1.0	0.8036	~2.42
ResNet50 + SepConv	1.0	1.0	0.75	0.923	1.0	0.714	1.0	**0.9045**	**~1.75**
	**F1 score of plants**							F1	
ResNet50	0.681	0.64	0.585	0.65	0.645	0.636	0.684	0.639	
ResNet101	0.614	0.597	0.585	0.591	0.614	0.636	0.684	0.619	
ResNet50 + SepConv	0.68	0.682	0.585	0.65	0.681	0.589	0.718	**0.652**	
	**MCC of plants**							MCC	
ResNet50	0.888	0.904	0.857	0.848	0.89	0.933	0.943	0.88	
ResNet101	0.799	0.844	0.852	0.794	0.924	0.914	0.929	0.86	
ResNet50 + SepConv	0.904	0.923	0.866	0.88	0.923	0.896	0.914	**0.89**	

From Figure 7, the 5th, 10th, 7th, and 22nd images showed the comparative performance of the backbone networks used in Mask R-CNN. The 7th and 22nd images had very sparse vegetation, but one with ripened plant parts, whereas 21st was a young green plant. All the metrics state that both networks can perform well if the vegetation has naked eye detectable spikes (7th and 22nd), and the performances of both of them get reduced a little in the case of mature plants due to the light texture of spikes (awns, hair, or bristle-like appendage get faded, and mask size reduces). The 5th (leaf interference) and 10th (overlapped spikes) images were typical plants with dense vegetation; ResNet50 showed a higher peak in all metrics than ResNet101. Therefore, ResNet50 would be performing better than ResNet101 for the early-stage spike detection and yield prediction.

5th, 6th, 10th, and 11th images primarily illustrate the effect of using a distinct convolutional technique, the separable convolution in the feature extraction blocks of the backbone. Two networks with ResNet50 and ResNet50 + SepConv obtained the same AP and F1 score in 5th and 10th images but later gave higher values in the case of the 6th and 11th images. The use of separable convolution showed better performance in the images with leaf interference (6th and 11th images). Replacement of standard convolution with separable convolution (SepConv) in the convolutional blocks backbone network (ResNet50) made more efficient use of model parameters and made an increase in mean precision (Chollet, 2017). Both ResNet50 and separable convolution lacked accuracy in sparse vegetation and light-colored texture, whereas ResNet101 got a better result. This is due to the detection of the more exact mask of spikes that is less than the ground truth so that it gets reduced values of all the metrics.

This comparative study resulted in the best network configuration for Mask R-CNN in spike detection. ResNet50 structure with separable convolution outperformed the other two networks (mAP of 90.45%, mean F1 value of 0.65, and MCC of 0.89 on the test dataset of 24 images). Also, it was observed to be suitable for spike detection with constraints like overlapping plant canopy, leaf interference, and variable illumination. ResNet50 made a light backbone network, and separable convolution enhanced the use of trained parameters that reduced the network complexity (ResNet50-179 Mb and ResNet101-256 Mb), and both training and inferencing time ResNet50 made a light backbone network that required less time at training and inferencing (1.75 s per image on CPU with 4392 BogoMibps) both.

#### Type of input data

Two types of the input dataset, i.e., RGB and HLS, were tested on the spike detection network. The basic model of Mask R-CNN (ResNet101 + Normal Convolution) was trained on these datasets. For evaluation, a new test dataset (seven images) was prepared to contain the plant images taken at different crop growth stages.

**Figure 8:**
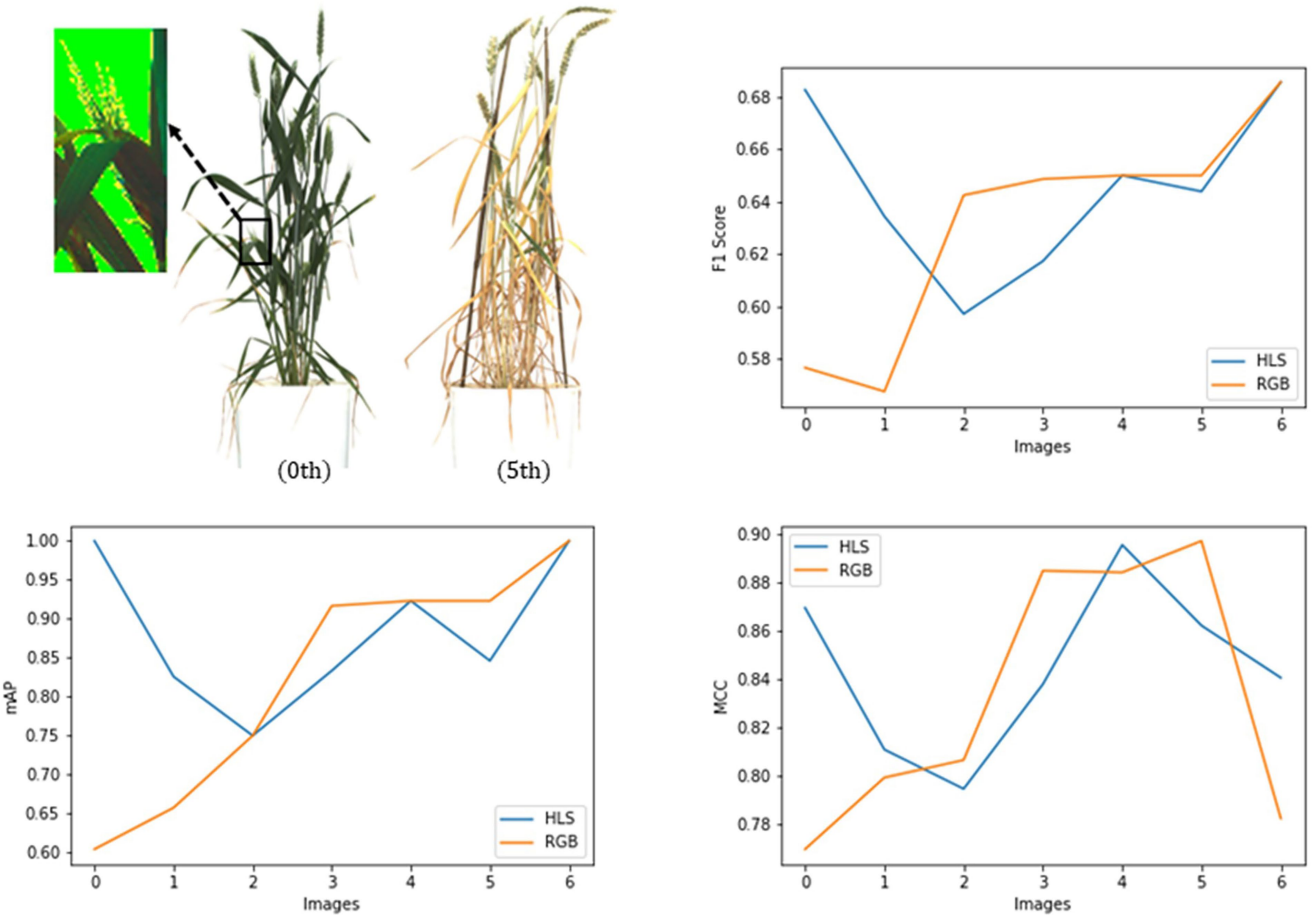
Evaluation of the Mask RCNN network on RGB and HLS image dataset with metrics, mAP, Fl score, and MCC.

Table 3 and Figure 8 state that in the case of a bushy young plant, i.e., overlapping spikes and leaves (0th plant), models detected spikes more precisely using HLS data. HLS data store the dominant color of the pixels along with the subsequent saturation and luminescent value. Spike edges showed highly variable reflectances to the light, which pursue good segmentation features (Intisar et al., 2021). But at the last stages of growth, leaves also turn yellow, and reflectance values of leaf and spike become nearly identical. So, the segmentation using HLS data cannot get good precision where the color features in RGB data can have (5th plant). The test dataset showed that the HLS format could enhance the mean average precision from 82.5% of RGB to 88.2% (with the F1 score of 0.65 and the MCC of 0.89). As the study mainly focused on the early-staged plants, the HLS was proved to be a better format for spike detection.

**Table 3:** Evaluation metrics of different trained models using RGB and HLS dataset.

Input data	Test plants		
	*a*	*b*	Mean
	Average precision (AP) of plants		mAP
RGB	0.604	0.923	0.825
HLS	1.0	0.846	**0.882**
	**F1 score of plants**		F1
RGB	0.576	0.65	0.631
HLS	0.682	0.643	**0.644**
	**MCC of plants**		MCC
RGB	0.769	0.896	0.832
HLS	0.869	0.862	**0.844**

Finally, the primary dataset trained the improved network structure (ResNet50 + SepConv + Input-HLS), and a set of well-mixed test images of plants obtained an mAP of 97.57%, a F1 score of 0.679, and an MCC of 0.911.

### Result of spike detection

The improved model dissected the images of wheat plants and took out the spikes (Figure 5). These images varied in shape and quality according to the input image of plants. Spike number and pixel areas were computed and passed to the yield prediction module (Table 4).

**Table 4:** Parameters of spikelet yield prediction from a sample plant.

Spike no.	Spike area	Number of spike-lets	Density (e^−4^)
1	9,286	8	8.6
2	**11,605**	11	9.4
3	9,583	9	9.3
4	10,227	13	12.7
5	9,966	20	20.06
6	8,861	18	**20.31**
7	6,880	11	15.9
8	7,121	10	14.04
9	5,908	11	18.6
10	5,489	2	3.6

### Evaluation of spikelet detection module

**Figure 9:**
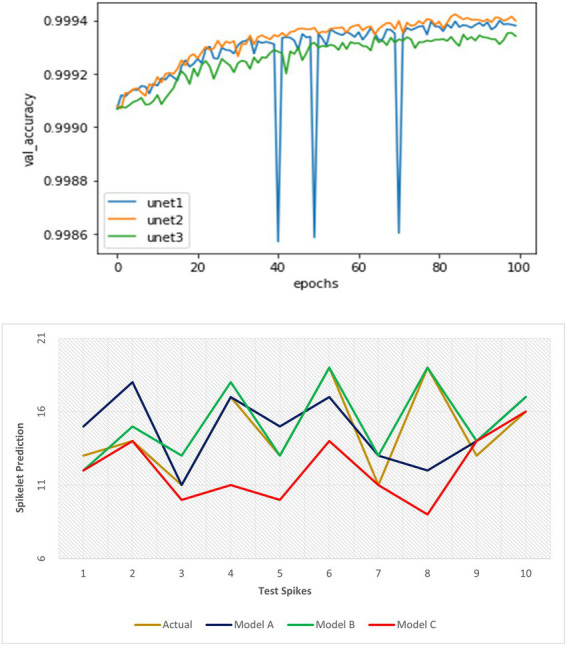
Performance evaluation of three spikelet detection networks using validation metrics at training and detection accuracy on test dataset respectively.

In the spikelet detection, all models obtained significantly fewer validation losses (~99% validation accuracy) for patches at certain depths of proposed U-Net networks. Further increase in the depth of our network was not required (Figure 9). The second model performed better in terms of validation accuracy and its consistency at detection (than the first network). Full binary images were created from the resulted patches, and a standard parameterized spikelet counting algorithm (a search for spikelet-sized binary objects, the optimum size varied according to the resolution of input images) was applied to get the number of spikelets in the spike.

Further evaluation of the models using three proposed parameters showed the true difference in detection performance (Table 5; Figure 9). The result illustrated that the first model could extract a precise feature map of objects at a good representation of spikelets and detected a good number of spikelets with less error outperforming the third model. However, in constraint cases, it suffers from undercounting where the second model could fetch better accuracy for all images of the spike dataset. Therefore, the second model was most efficient and consistent considering its mSE (1.3) as well as scoring of valid over-detection (more OC) and least under-detection (UC).

**Table 5:** Evaluation of spikelet detection models.

Spikes	Actual	Model A	Model B	Model C
1	13	15	12	12
2	14	18	15	14
3	11	11	13	10
4	17	17	18	11
5	13	15	13	10
6	19	17	19	14
7	11	13	13	11
8	19	12	19	9
9	13	14	14	14
10	16	17	17	16
OC/UC (mSE)		6, 3 (8.3)	6, 1 (1.3)	1, 6 (17.3)

The loss function was good in assessing the proposed architectures of the network, that is, U-Net, whereas the latter drew the best model out of the U-Net models.

### Result of spikelet detection

Spikelet detection took each spike image of a plant, and the number of spikelets was obtained (Table 4) that would be used in the yield prediction.

### Spikelet yield prediction

For evaluating the yield prediction methodology, a sample plant was taken with 10 spikes, and the previous modules gave desired outputs used in Table 4.

The density of spikelets in spikes (spikelet number per spike area) was preferred as an authentic parameter to compute spikelet yield. Maximum density observed among all spikes denoted true spikelet bearing potentiality in each spike. The second parameter, the maximum spike pixel area, was the spike area from the best-observed spike. Both represented the common property of spikes in the plant leading to a precise yield estimate (better result than the direct spikelet counting method).

Putting max spike area and max density (Table 4) as 11605 and $20.31e^{-4},$ respectively, in Equation 8, we get


$$\mathrm{SY}=11605*20.31e^{-4}*10≅236$$

## Discussion

**Figure 10:**
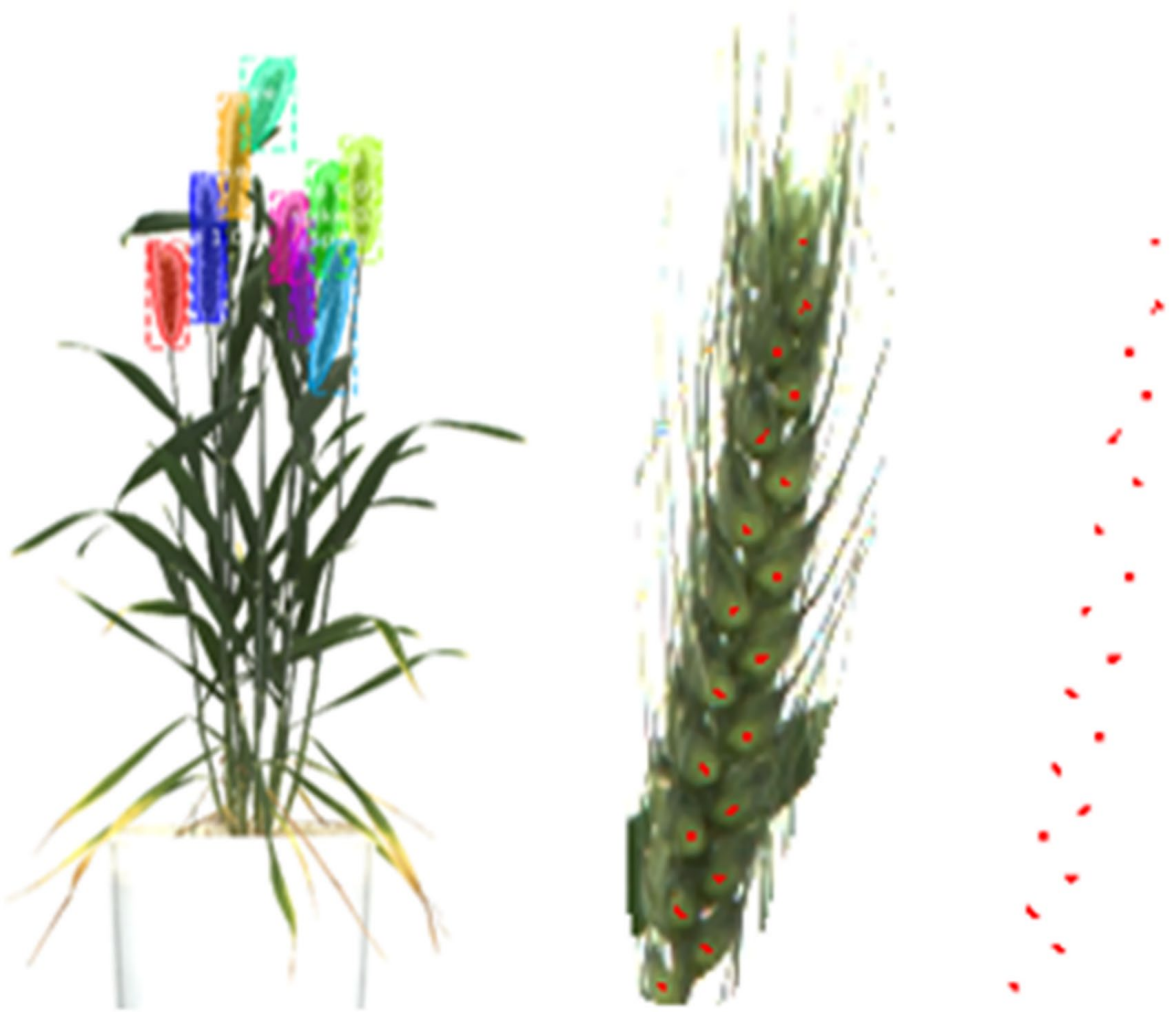
The result of the proposed methodology, SlypNet.

This study presented a novel and precise spikelet-based yield prediction methodology from the wheat plant’s visual images (Figure 10) using a deep learning architecture comprising enhanced models of Mask R-CNN and U-Net. From the plant to spikelet, an unbiased, multistage and visionary approach of artificial intelligence (AI) and optimistic mathematics predicted the spikelet yield, an estimate of grain yield without using any auxiliary growth parameter.

In wheat, the existing visual techniques mainly used two ways to predict crop yield. First, the number of produced spikes can predict an estimate of crop yield (Hasan et al., 2018; Misra et al., 2020). In the latter, direct quantification of the spikelet number can make a more accurate yield prediction (Alkhudaydi and Zhou, 2019). Technology advancement came up with several object detection models of deep learning that precisely detect the spikes and spikelets from plant images. All detection techniques obtained a certain accuracy level and faced some questionable constraints for the images. So, these constraints always encouraged data scientists to build more precise and adaptable models.

In spike detection, several CNN architectures like R-CNNs, YOLOs, and U-Nets have been used to make automatic visual detection of spikes. This spike led to yield prediction also, which meets the demand for standing crop yield assessment in the wheat crop. These techniques gained good accuracy, but the precision changes due to their own functional limitation as to the model changes. R-CNN and YOLO can give an accurate number of spikes but not the precise features of an individual spike. The segmentation network, U-Net, can obtain good feature extraction of each spike but requires an additional counting algorithm for getting the number of spikes needed in yield prediction. Besides the structural constraints of models, their performance got limited in many cases of real plant images. Overlapped leaves and spikes, various illumination, image resolution, and background interference are present in the images collected from different stages, variety, and illuminized conditions. Therefore, there is a need to upgrade existing methodologies and build up a more precise and robust spike detection technique.

Yield prediction based on the spikes will make a reasonable yield estimate but is not as accurate and unbiased as spikelet-based yield prediction. Due to genetic and anatomical variation, the number of spikelets in a spike varies from plant to plant so that the plants with an equal number of spikes might not result in the exact yield, but the existing spikelet detection techniques do not provide that much accuracy. The resulted outcomes from them are obsolete in most cases. For example, the direct detection of spikelets without segmenting the spikes might detect false spikelets due to the presence of diseased spots on the leaves homologous to spikelets (overcounting), and variation in the orientation of spikes could not detect the full number of spikelets from each spike which might cause the lesser estimate of yield (undercounted). This malfunction was due to the use of a full-plant body and its low-resolution image. So, spikelet detection could perform better if done only after detecting the crop reproductive part (spike in wheat). Therefore, a new redesigned architecture of yield prediction, SlypNet, has been proposed in this study that deploys two upgraded novel modules, that is, spike detection followed by spikelet detection.

To detect the spikes from a plant for the first time, an instance segmentation architecture, Mask R-CNN, was applied to increase precision and overcome many constraints faced in the previous spike detection methods. In spike detection, instead of the bounding box, the mask makes a more accurate segmentation of spikes (Su et al., 2021) by avoiding background interferences that make the further morphological feature analysis more reasonable. Separate masks gave instant segmentation of spikes that improved spike detection in several ways. It gave instances of individual spike objects (the bounding boxes), reduced the burden of additional spike counting algorithms, unlike U-Nets (Misra et al., 2020), and also more clear pixels belonging to individual objects so that each mask could provide accurate pixel area of spikes, unlike bounding boxes in other R-CNNs (Hasan et al., 2018). Variable-sized anchor boxes in RPN solved the problem of feature extraction from various resolutions of input images. So, the use of Mask R-CNN in the spike detection overcame many of the previously faced network constraints.

Subsequent parameter tuning and structural changes in the backbone of the benchmark network of Mask R-CNN gave a precision-optimized and light-weighted network that can make superior spike detection in other constraints also. ResNet50 in place of denser ResNet101 reduces the complexity and makes a light network that takes less training and inferencing time. This light structure network trains its parameters more rationally. It extracts a more precise feature that enhances the network’s performance in the images with constraints like overlapped spikes and leaves. Further reform in the core convolution technique, i.e., the use of separable convolution, drew more precision by using the trained parameters more efficiently and conquered the constraints, leaf interference in spike detection. Besides the structural modifications, change in the input data type also helped. HLS data give more detectable features than RGB in the image of early-stage young plants. So, the significant constraints like highly bushy green vegetation (spike to spike overlapping) (Misra et al., 2020) also get higher precision by using HLS type data during spike detection. The new and updated network (ResNet50 + SepConv + HLS) was trained on the images with every kind of constraints, and an improved data-specific architecture of Mask R-CNN (97.57% mAP, 0.91 MCC, and 0.67 F1 score) in the spike detection was developed for the standing crop yield prediction.

The spikelet detection network used an improved architecture of the U-Net model that can generate a highly determinate feature map of spikelets in the spike. The model evaluation provided the best performing spikelet detection network with very less error (99% validation accuracy and 1.3 MSE in the test dataset). This spikelet detection model also gets a better result as it takes only the spike images, unlike spikelet detection by Alkhudaydi and Zhou (2019).

All the detected visual features from the plant, spike, and spikelets modeled the potential yield of the crop plant. So, SlypNet excels other methodologies in terms of robustness and unbiasedness in the computation of yield as it considers both the spike and spikelet and by deploying the latest and upgraded architectures of deep learning in the spike and spikelet detection.

This study gets high precision in spikelet-based yield prediction, but spikelet is not the ultimate yield quantifier. Grains are present in the spikelets’ awns, so they cannot be observed in the images (Supplementary Figure S6). Awns number in each spikelet varies, so the exact grain yield cannot be predicted or mathematically modeled from spikelet yield. So the spikelet-based yield gave us the number of produced spikelets, not the weighted measure of yield. This study also faced little error in spikelet yield prediction due to only 2D views of plants, so it needs 3D modeling of plants that may provide a more accurate spikelet yield.

However, SlypNet generated a digital platform for diagnosing growing plants and predicting the yield potentiality using visual aids with significant precision.

## Conclusion

In this proposed methodology, SlypNet, a package of deep learning and image processing techniques, paved the way to obtain the true yield of the wheat plant. Plant to spikelet, a two-stage deep learning methodology, improves yield prediction and develops new spike and spikelet detection networks. Not only the yield prediction but also each module was improved with great precision. Spike detection got a new data-driven deep learning model, Mask R-CNN (97.57% mAP, 0.91 MCC, and 0.67 F1 scores), that can tackle a major number of constraints faced by previous spike detection models and proved itself better for the precise yield prediction also. A subsequent feature analysis gets its enriched output and builds an explicit spikelet detection model (99% validation accuracy and 1.3 MSE in the test dataset). Modularization helped with more accuracy and consistency in the yield prediction in various constrained situations. Improved object detection networks extend the scope of implementation since detected spikes and spikelets generated from the networks can provide us with more physiological information about the spike, spikelet, and leaf. Since the networks were trained with all possible plant images collected at different growth stages of the spike, it helps in decision-making throughout the growing period. The absence of auxiliary parameters and feasibility with images makes this technique instant, automated, and less expensive. The spikelets produced in a plant, not the weighted grain yield, gave us the predicted spikelet yield. So, further analysis of the anatomical behavior of spikelets and seeds present in it and the weight measurement can give us the actual yield of wheat and other crops.

## Data availability statement

The raw data supporting the conclusions of this article will be made available by the authors, without undue reservation.

## Author contributions

AM: design the methodology and paper writing. SM and VC: conceptualization. SK: methodology and objective refinement. AA and SI: draft correction and resources support. All authors contributed to the article and approved the submitted version

## Conflict of interest

The authors declare that the research was conducted in the absence of any commercial or financial relationships that could be construed as a potential conflict of interest.

## Publisher’s note

All claims expressed in this article are solely those of the authors and do not necessarily represent those of their affiliated organizations, or those of the publisher, the editors and the reviewers. Any product that may be evaluated in this article, or claim that may be made by its manufacturer, is not guaranteed or endorsed by the publisher.

## Supplementary material

The Supplementary Material for this article can be found online at: https://www.frontiersin.org/articles/10.3389/fpls.2022.889853/full#supplementary-material
