# Supplementary figures and images for: SlypNet: Spikelet-based yield prediction of wheat using advanced plant phenotyping and computer vision techniques
Source: Front Plant Sci. 2022 Aug 4;13:889853. doi: 10.3389/fpls.2022.889853 (PMC9386505; doi:10.3389/fpls.2022.889853)

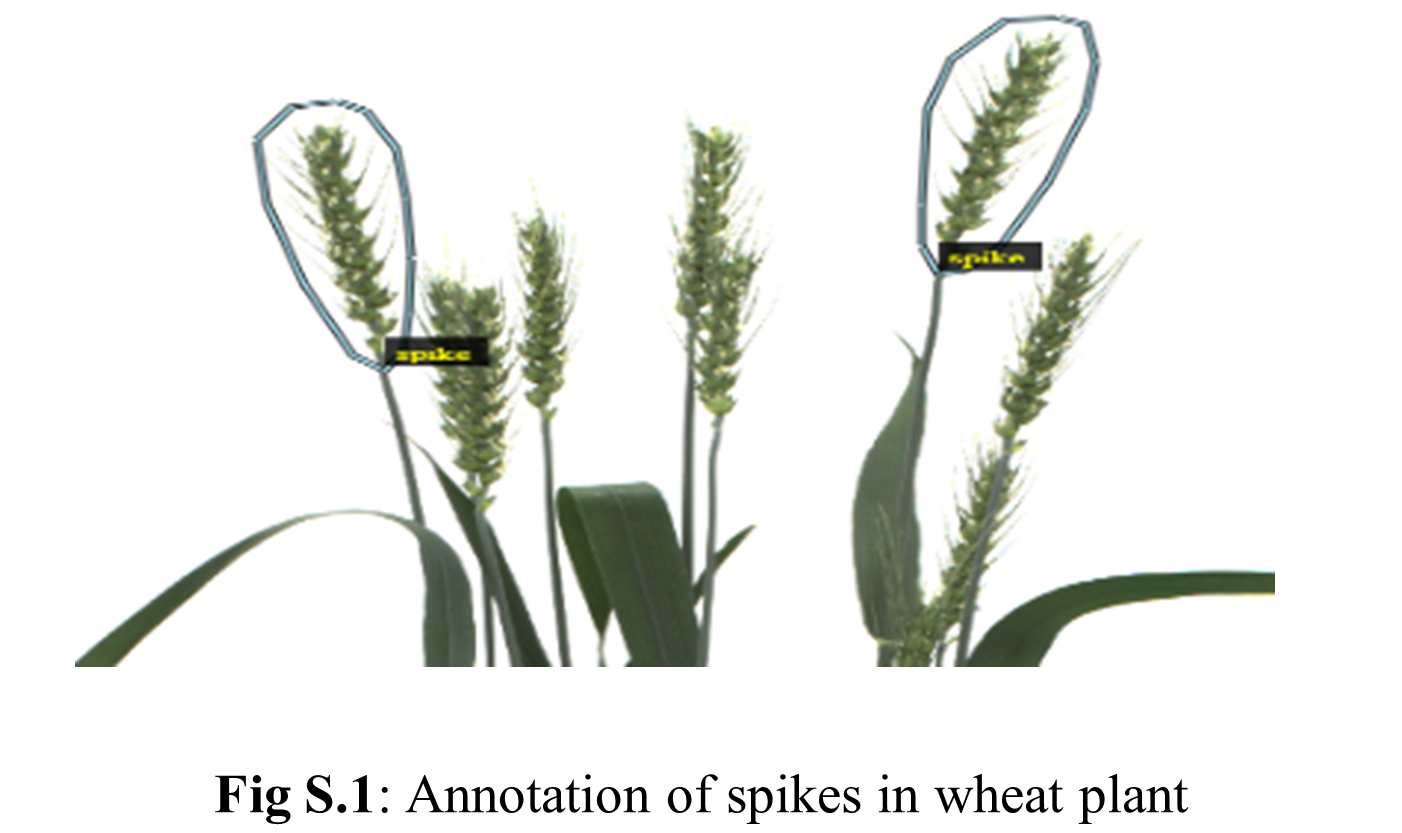

Supplement: Supplementary file 1 [file Image_1.JPEG]

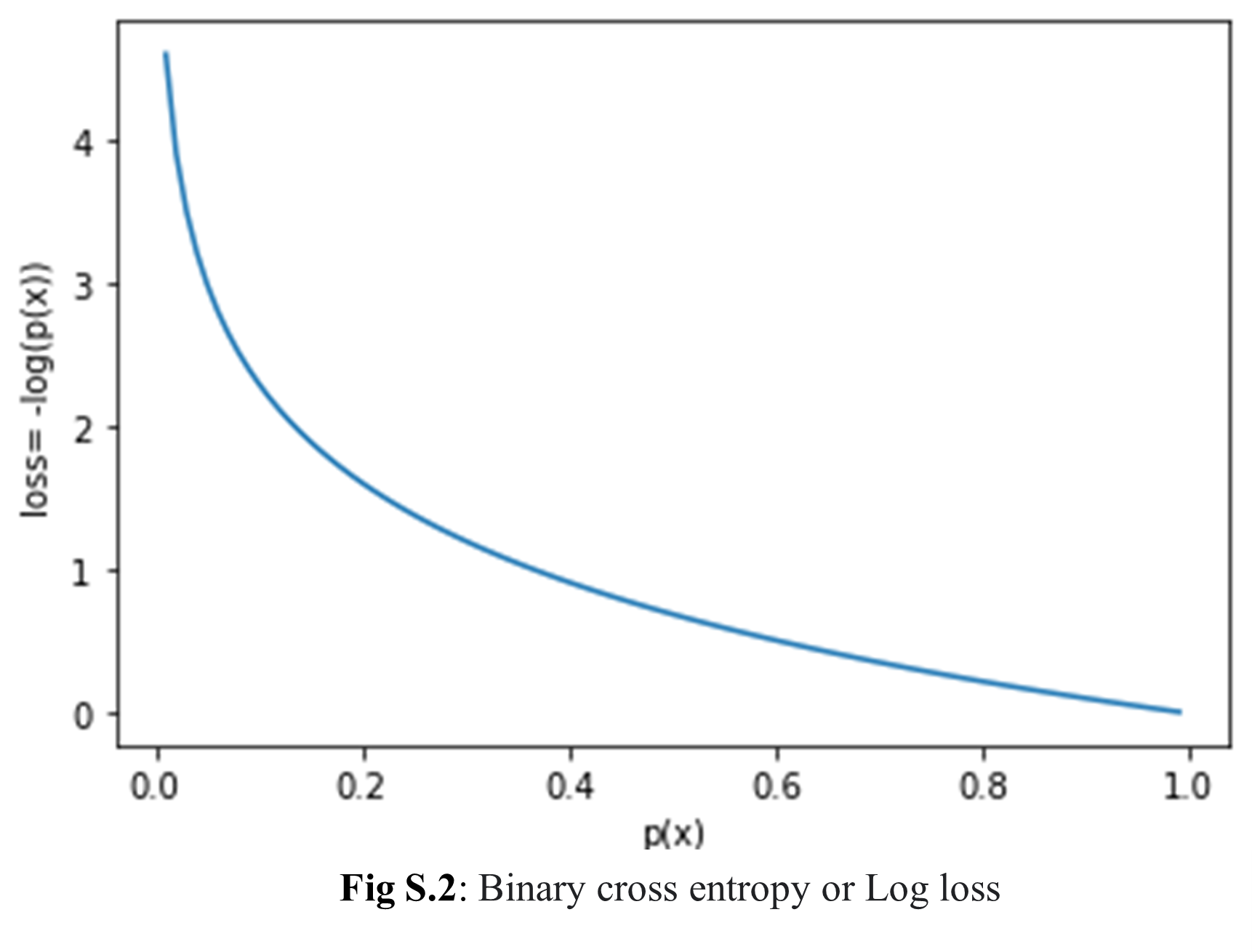

Supplement: Supplementary file 2 [file Image_2.JPEG]

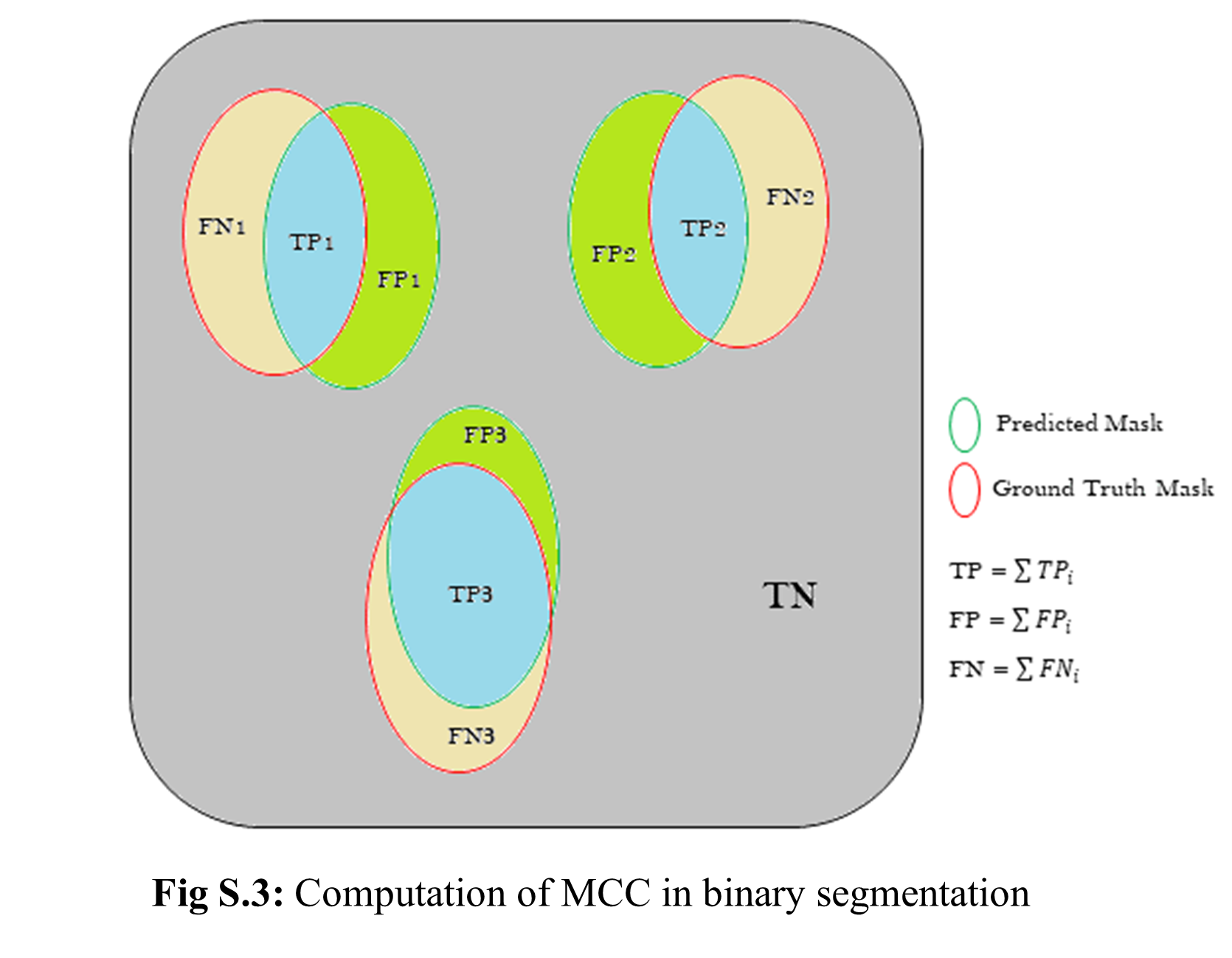

Supplement: Supplementary file 3 [file Image_3.JPEG]

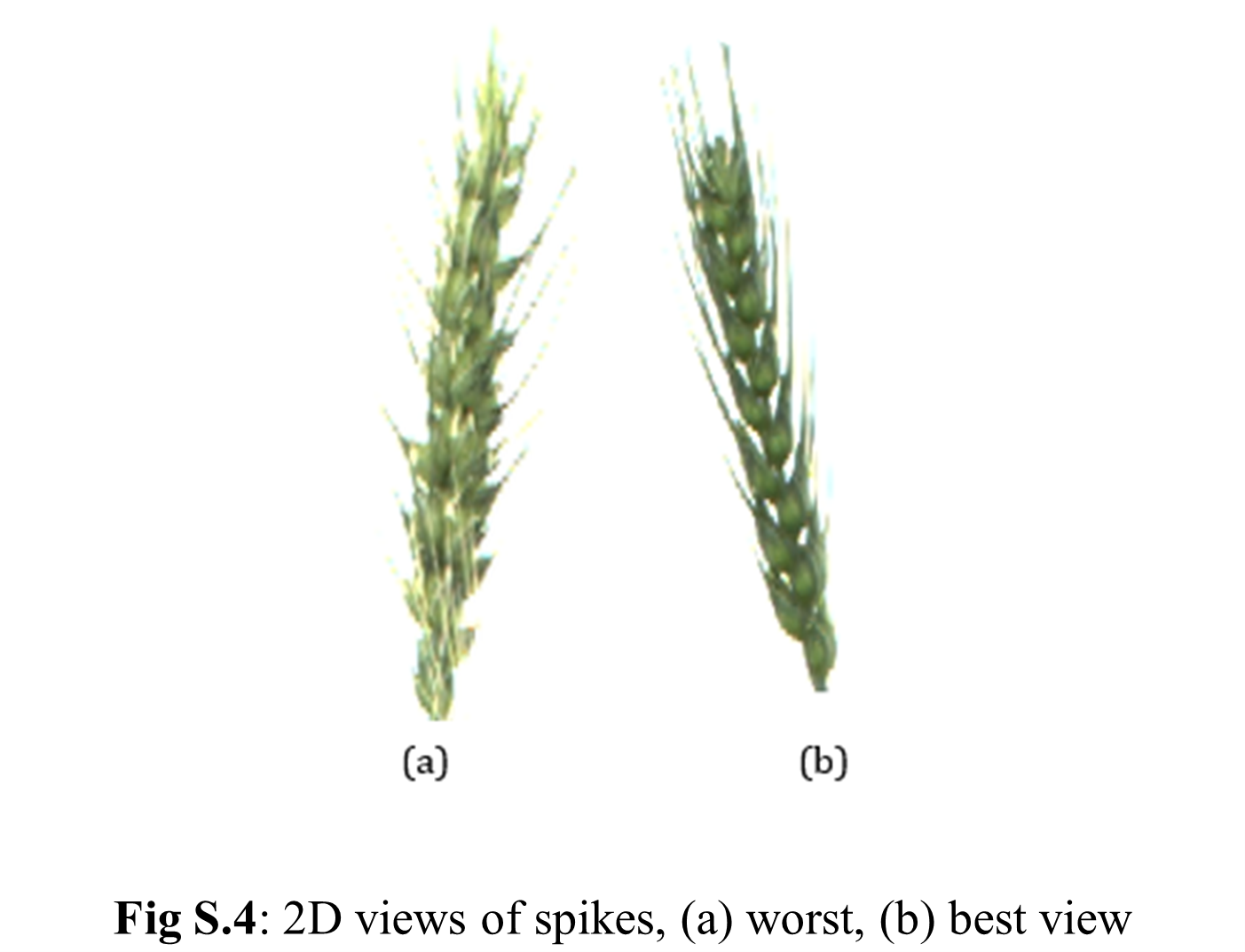

Supplement: Supplementary file 4 [file Image_4.JPEG]

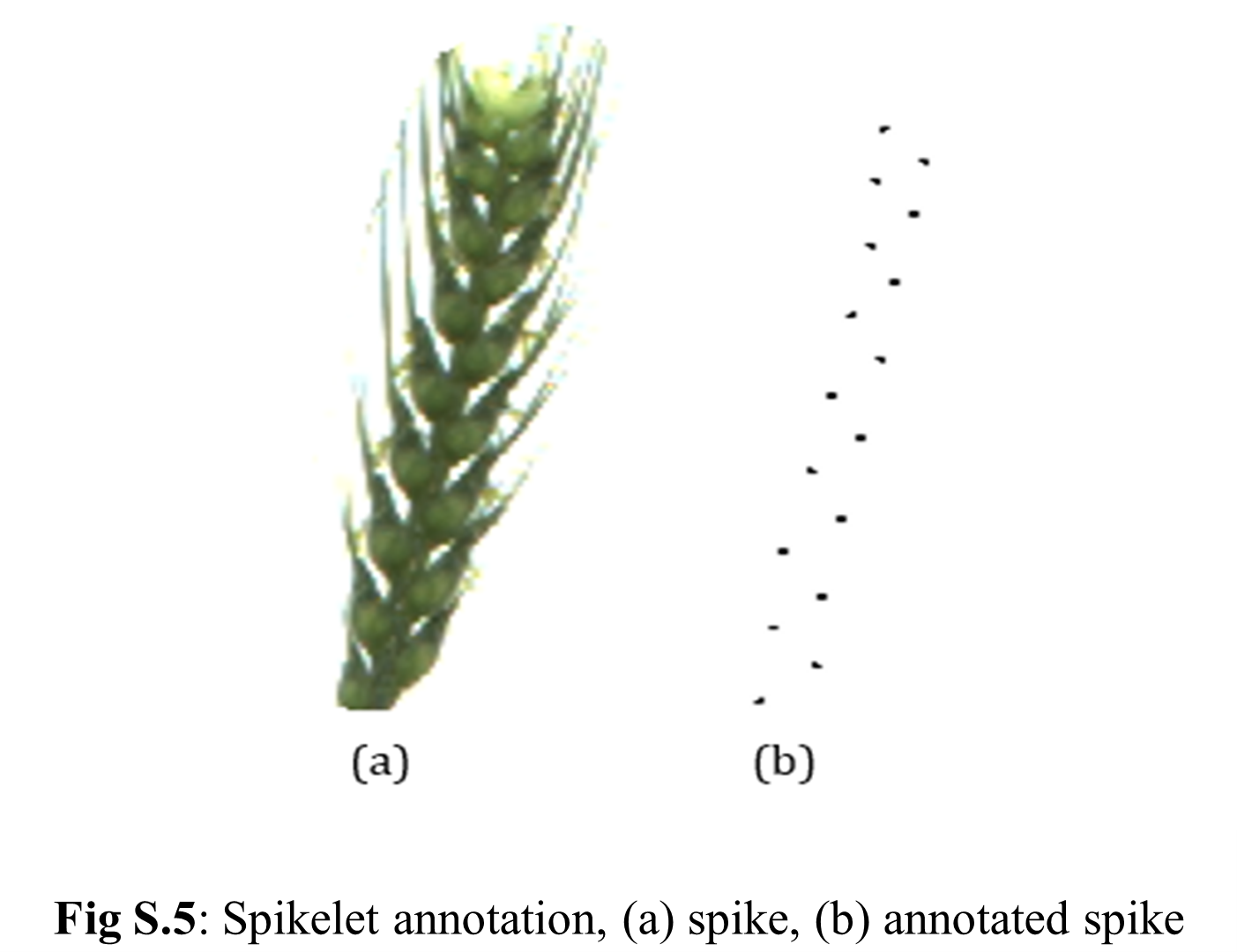

Supplement: Supplementary file 5 [file Image_5.JPEG]

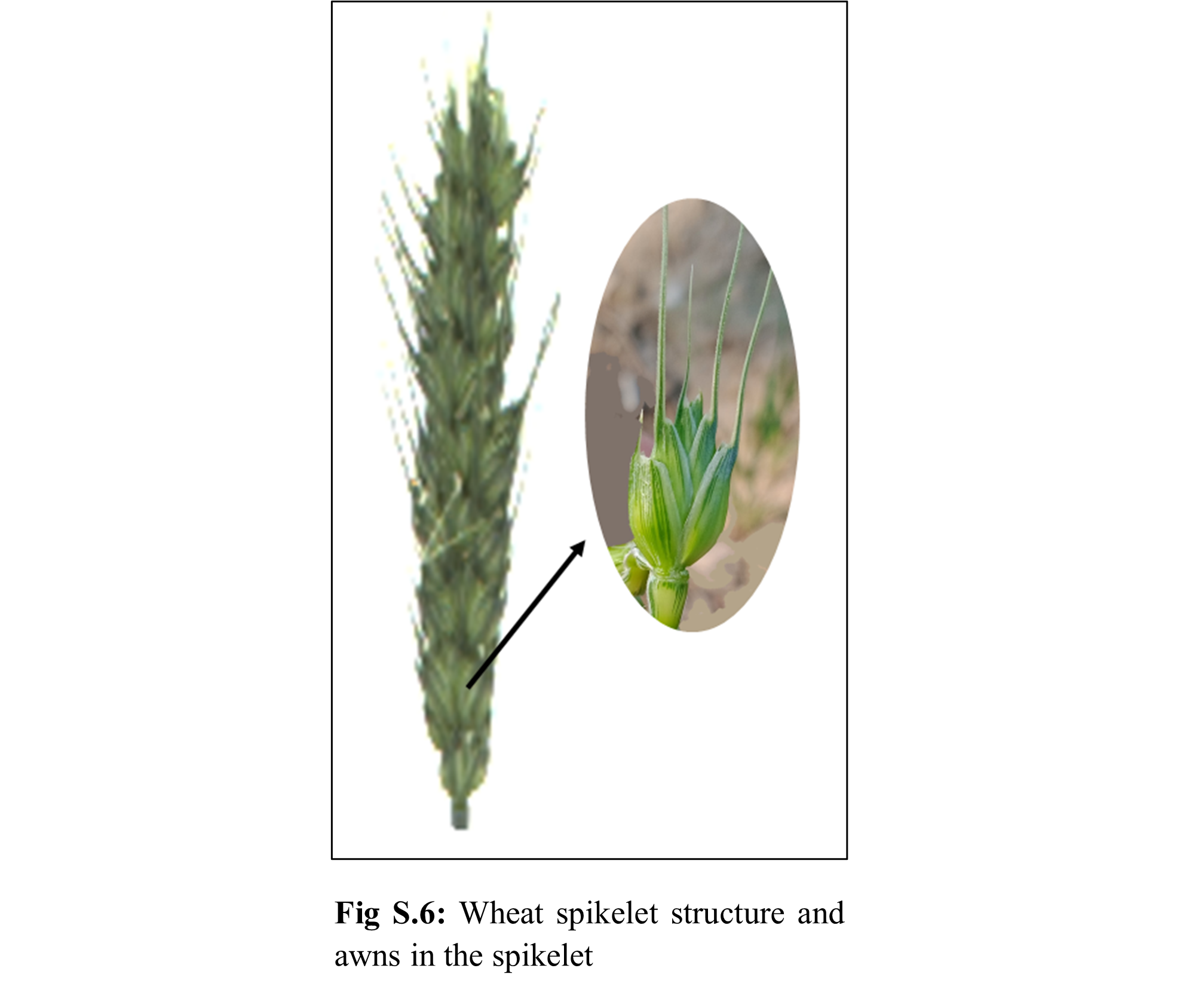

Supplement: Supplementary file 6 [file Image_6.JPEG]
